# CfDNA as a surrogate marker for COVID-19 severity in patients with influenza-like symptoms with and without SARS-CoV-2 infection in general practice: a prospective cohort study

**DOI:** 10.1186/s12985-025-03016-x

**Published:** 2025-12-01

**Authors:** Dorothea Dehnen, Suzan Botzenhardt, Ekaterini Giagkou, Kira Enders, Katharina Hoeter, Perikles Simon, Elmo W.I. Neuberger

**Affiliations:** 1 Institute of General Practice, Medical Faculty, University of Duisburg-Essen, Essen, Germany; 2 West German Cancer Center, University Hospital Essen, Essen, Germany; 3 Department of Sports Medicine, Rehabilitation and Disease Prevention, Johannes Gutenberg University of Mainz, Mainz, Germany; 4 Department of Anaesthesiology, University Medical Centre of the Johannes Gutenberg- University, Mainz, Germany

**Keywords:** SARS-CoV-2 infection, Cell-free DNA (cfDNA), Flu/influenza, Predictive marker, General practice

## Abstract

**Background:**

Cell-free DNA (cfDNA) has emerged as a relevant biomarker reflecting disease severity in hospitalised COVID-19 patients, correlating with respiratory failure and mortality. However, its utility has not yet been evaluated in general practitioner setting.

**Methods:**

A prospective single-centre, two-arm, parallel, longitudinal cohort study conducted in a German general practice with four doctors between 8/2021 and 4/2022. *Participants*: Sixty-one outpatients with flu-like symptoms participated: 31 (10 men, 21 women) tested SARS-CoV-2 positive (COVID group); 30 (12 men, 18 women) were controls (control group). The groups were demographically similar. *Primary outcome measures*: Comparison of cfDNA levels between groups at day 0, 7 and 14. *Secondary outcome measures*: Correlations between cfDNA levels and laboratory and clinical parameters like blood counts, respiratory rate and oxygen saturation.

**Results:**

cfDNA levels did not differ significantly between groups (F [1, 59] = 1.538, *p* = 0.22): day 0: mean (± standard deviation) = 14.45 (± 6.24) ng/ml (COVID group) vs. 11.32 (± 4.79) ng/ml (control group); day 7: 14.46 (± 6.57) ng/ml vs. 12.53 (± 6.67) ng/ml; day 14: 12.94 (± 6.66) ng/ml vs. 12.93 (± 7.02) ng/ml. However, at t0, the integrity index was significantly lower in the COVID group (t0: 0.30 [±- 0.15] vs. 0.41 [± 0.1]; *p* = 0.0127) increasing at t1 (0.38 [± 0.17]; *p* = 0.008) and at t2 (0.42 [± 0.22]; *p* < 0.001).

**Conclusion:**

Unlike hospitalised patients, cfDNA levels did not differ significantly between outpatient groups. Therefore, a decision on the need for hospitalisation based on clinical and serological factors is still required. The significantly lower integrity index of the SARS-CoV-2 infected individuals indicates that their DNA kinetics differ from those of individuals infected with other respiratory pathogens.

**Trial registration:**

: German Clinical Trials Register: DRKS00024722, Registration date: 10 March 2021.

**Supplementary Information:**

The online version contains supplementary material available at 10.1186/s12985-025-03016-x.

## Introduction

Since 5 May 2023, more than three years after the start of the COVID-19 pandemic, it is no longer considered an international health emergency by the WHO [1]. Nevertheless, SARS-CoV-2, particularly the Omicron subvariants, remain a dominant cause of winter respiratory infections, alongside seasonal respiratory pathogens such as influenza, human seasonal coronaviruses, human metapneumoviruses, rhinoviruses and respiratory syncytial virus (RSV) [2, 3]. The clinical presentation of COVID-19 varies wildly, ranging from asymptomatic or mild respiratory symptoms to severe disease requiring hospitalisation, intensive care, or resulting in death [4–6]. Although Omicron has a reduced fatality rate compared to earlier variants like Delta, it is more transmissible and can still cause severe outcomes, especially in elderly, immunocompromised or unvaccinated individuals [6, 7]. A large-scale UK cohort study confirms that even among vaccinated individuals, those with immunosuppression, multimorbidity (*≥* 5 comorbidities) and advanced age (> 80 years) remain at increased risk of severe outcomes [8]. A recent Danish study (2022–2024) shows that the Omicron disease burden remains higher than that of influenza in terms of hospitalisations, complications and mortality, especially among unvaccinated men with co-morbidities [9]. Studies on vaccine effectiveness showed a lower effectiveness of mRNA vaccines against Omicron variants due to immune escape mechanisms [10–12]; however, booster vaccinations improve protection against severe illness [13–15]. Despite this, vaccination uptake remains low: for example, only 16% of Germans over 60 years received the recommended COVID-19 booster in 2022/2023 [16].

Consequently, vulnerable groups will continue to require special protection strategies in the future, especially with increasingly infectious variants. Early use of antiviral agents like Paxlovid^®^ can mitigate disease severity [17], yet reluctance among general practitioners – due to uncertainty about their application and concerns about drug interactions or rebound effects [18] – often delays or prevents timely treatment. A reliable blood-based biomarker to identify high-risk patients early, regardless of vaccination status, could help guide early intervention, lower the threshold for antiviral prescription and ensure timely hospital referrals.

One promising biomarker is cell-free DNA (cfDNA), which enters the bloodstream during cellular apoptosis, necrosis, NETosis and active secretion [19, 20]. Elevated cfDNA levels correlate with disease activity in cancer and autoimmune disorders [21–23]. In both influenza and COVID-19, cfDNA levels have been reported as a prognostic biomarker in predicting disease progression and clinical outcome [24–27].

Importantly, not only the cfDNA level but also its fragmentation pattern, quantified by the integrity index, provides insights into the mechanism of cell death (e.g. apoptosis vs. necrosis) and tissue of origin. This makes the integrity index a potentially valuable parameter for understanding the biological impact of infection beyond total cfDNA levels [28]. Previous studies have focused mainly on hospitalised patients with moderate to severe COVID-19 [26, 27, 29]. In contrast, our study focuses on outpatients with flu-like symptoms treated in general practice who did not require hospitalisation. The objective was to determine whether cfDNA levels and the integrity index differ between patients with a confirmed SARS-CoV-2 infection and those with other respiratory viruses.

## Methods

### Design and patients

This is a prospective single-centre, two-arm, parallel cohort study with a 1:1 allocation ratio, which was conducted at the University Hospital in Essen (Germany) between August 2021 and April 2022. Ethical approval was granted by the Ethics Committee Essen (No. 21–9916-BO). Patients were recruited before the Omicron variant emerged in Germany, during a period when the Delta variant was dominant [30]. It is therefore assumed that all SARS-CoV-2 cases were Delta infections. During recruitment influenza activity remained low, with < 5% of samples testing positive according to the Influenza Working Group of the Robert Koch Institute [3].

Adults aged ≥ 18 years presenting to their general practice (GP) in Mülheim/Ruhr, Germany, with flu-like symptoms were eligible. After informed consent, blood samples and nasopharyngeal swabs were collected. COVID-positive patients were visited at home for study procedures. Exclusion criteria included any known acute or chronic illness (e.g. tumour disease, severe renal insufficiency, severe/moderate inflammatory disease, autoimmune disease, rheumatological disease) associated with elevated cfDNA levels.

### Study procedure

On the day of presentation, patients underwent a SARS-CoV-2 rapid antigen test (Roche SARS-CoV 2 Rapid Antigen Test) followed by RT-PCR. Based on the result, patients were assigned to either the group “SARS-CoV-2 positive with flu-like symptoms” (COVID group) or to the group “SARS-CoV-2 negative with flu-like symptoms” (control group). No randomisation or blinding was applied.

Venous blood samples (15.3 mL) were collected at three points in time (t0 = day of recruitment, t1 = after 7 days, t2 = after 14 days) to determine cfDNA levels and inflammatory markers (C-reactive protein [CRP], erythrocyte sedimentation rate [ESR] and a differential blood count). Clinical parameters such as temperature, oxygen saturation and respiratory rate were recorded and patients rated current symptom severity on a Likert scale (0: no symptoms, 10: strongest symptom intensity) [31]. Blood was centrifuged at 1600 ×g and stored at −18 °C in the GP practice, then sent temperature-controlled to the Department of Sports Medicine, Prevention and Rehabilitation at the Johannes Gutenberg University Mainz. There, samples were centrifuged again (16,000 ×g for 10 min) prior to a protocolled qPCR analysis [32].

After six months, patients in the COVID-19 group were contacted to assess for long-term post-COVID-19 syndrome according to the NICE guidelines [33]. In symptomatic individuals, cfDNA levels were remeasured.

### Measurement of CfDNA levels

cfDNA was quantified in an S2 laboratory at the Department of Sports Medicine, Prevention and Rehabilitation at the Johannes Gutenberg University Mainz, using validated qPCR assays targeting 90 bp and 222 bp fragments in diluted EDTA plasma, without prior DNA isolation [32]. The integrity index was calculated as the ratio of long (222 bp) to short (90 bp) fragment concentrations. All measurements were blinded and performed with the Assist Plus automated pipetting system (INTEGRA Biosciences, Switzerland). Full assay details are provided in the study protocol [34].

### Outcomes

The primary outcome was the comparison of cfDNA levels in the COVID group and the control group at t0 (day 0), t1 (day 7) and t2 (day 14), using self-established qPCR assays.

The secondary outcome was to analyse the correlation between cfDNA levels and laboratory parameters (e.g. haemoglobin, leukocytes and differential blood count), clinical parameters (e.g. blood pressure, pulse and oxygen saturation) and symptom severity.

### Statistical methods

Statistical analysis was conducted using R (Foundation for Statistical Computing, Vienna, Austria) [35]. Descriptive statistics were used to characterise both groups at baseline; group differences for risk factor variables were analysed using Fisher’s exact test.

Group differences over time were analysed using mixed ANOVA with Bonferroni corrected post-hoc t-tests (statix package version 0.7.2). Data were log-transformed and tested for normality using the Shapiro-Wilk test. The Levene test was applied to test for homogeneity of variance. For non-normally distributed parameters (diastolic blood pressure, CRP, ESR, oxygen saturation, absolute basophil count and absolute eosinophil count), non-parametric ANOVA was used (ARTool package, version 0.11.1), followed by Bonferroni corrected Wilcoxon rank-sum tests. Wilcoxon signed-rank tests were also used to describe differences in clinical symptoms. Spearman rank correlation coefficients were calculated to explore relationships between cfDNA levels and laboratory/clinical parameters. In the control group, nine patients dropped out; seven were excluded after testing positive for SARS-CoV-2 during follow-up, potentially biasing cfDNA levels. Due to the potential confounding effect of COVID-19 infections in the control group, the intention-to-treat analysis was omitted. P-values < 0.05 were considered statistically significant.

### Patient and public involvement

Patients or the public were not involved in the design, or conduct, or reporting, or dissemination plans of our research.

## Results

### Study cohort

**Fig. 1:**
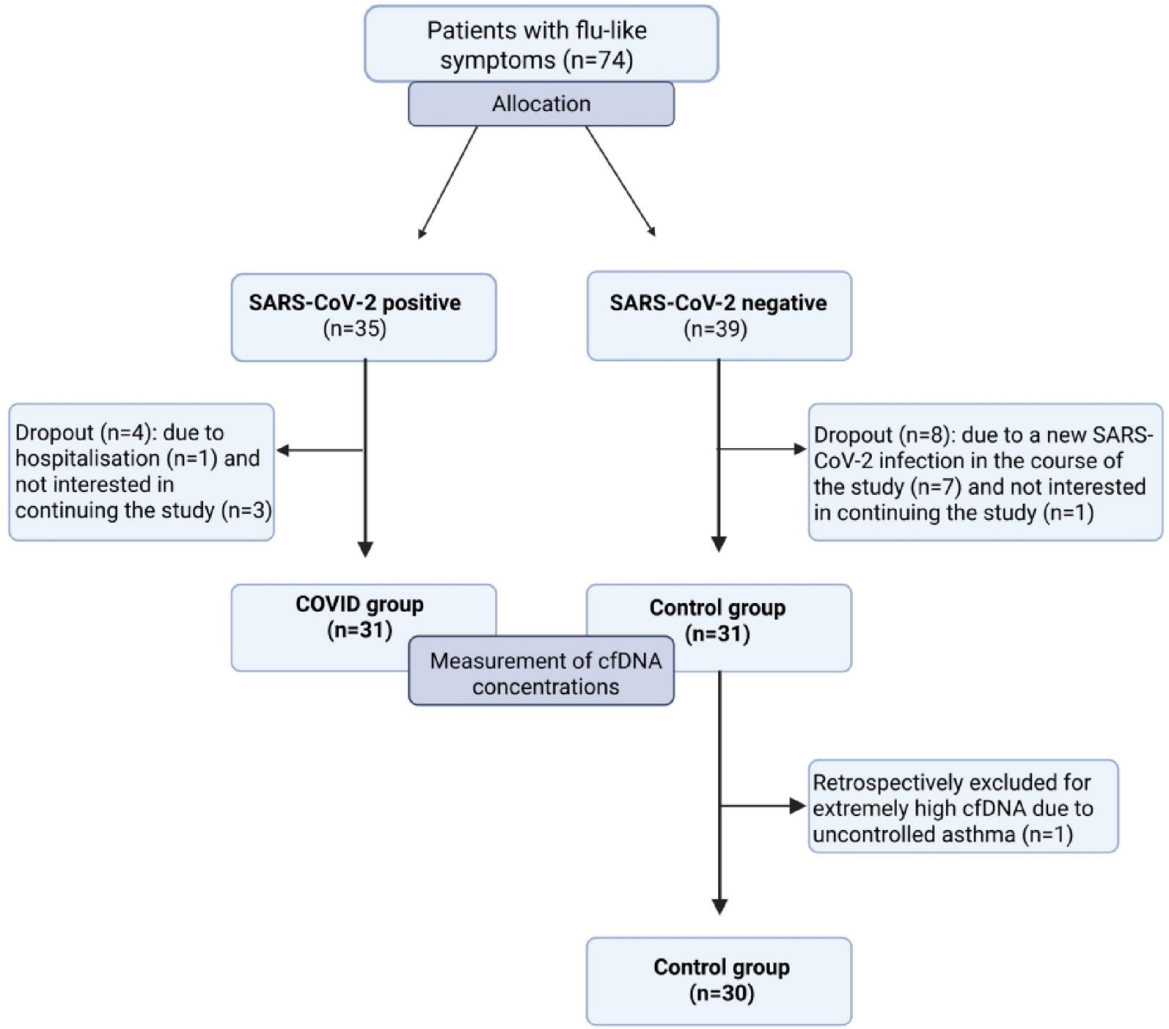
CONSORT flow diagram, created in BioRender

A total of 74 patients (35 in the COVID group and 39 in the control group) who met the inclusion and exclusion criteria were recruited in Mülheim/Ruhr, Germany. After dropout, data were analysed for 31 patients in the COVID group and 30 patients in the control group. For more details see Fig. 1.

All patients were classified as having ‘ambulatory mild disease’ according to the WHO Clinical Progression Scale [36]. The characteristics of the COVID and control group were similar: the COVID group consisted of 31 patients (10 men and 21 women) with a mean age of 39 years, the control group of 30 persons (12 men and 18 women) with a mean age of 38 years. The proportion of people with overweight (26.7% vs. 9.7%) or obesity (10.0% vs. 3.2%) was higher in the control group. In contrast, there were slightly more smokers in der COVID group (35.5% vs. 26.7%). Few people in both groups had pre-existing medical conditions. None of the people in the COVD-19 group reported symptoms of post-COVID-19 syndrome when contacted again after six months. For more details see Table 1.

**Table 1:** Comparison of the sample characteristics of the two groups using fisher’ exact test or Chi-squared test

Variable	COVID (*n* = 31)	Control (*n* = 30)	*p*-value^#^
Age in years (SD)	38.7 (± 13.5)	38.2 (± 16.3)	
Gender, n (%)			
Men	10 (32.2)	12 (40.0)	
Women	21 (67.7)	18 (60.0)	
Risk factors, n (%)			
Smoking	11 (35.5)	8 (26.7)	0.64^##^
Overweight	3 (9.7)	8 (26.7)	0.11
Arterial hypertension	2 (6.5)	3 (10.0)	0.67
Obesity	1 (3.2)	3 (10.0)	0.35
Bronchial asthma	2 (6.5)	2 (6.7)	1
Pregnancy	1^1^ (3.2)	0 (0)	1
Obstructive sleep apnoea syndrome	1 (3.2)	0 (0)	1
Type 2 diabetes mellitus	1 (3.2)	1 (3.3)	1
Coronary artery disease	0 (0)	1 (3.3)	0.49

^1^11th week; ^#^ using Fisher’s exact test; ^##^ using Chi-squared test

### Comparison of plasma CfDNA levels and integrity index between the two cohorts

Plasma cfDNA levels did not differ significantly between the two cohorts (F [1, 59] = 1.538, *p* = 0.22): day 0: mean (± standard deviation) = 14.45 (± 6.24) ng/ml (COVID group) vs. m = 11.32 (± 4.79) ng/ml (control group); day 7: m = 14.46 (± 6.57) ng/ml vs. m = 12.53 (± 6.67) ng/ml; day 14: m = 12.94 (± 6.66) ng/ml vs. m = 12.93 (± 7.02) ng/ml. There was a significant interaction in the integrity index (F [1.76, 103.93] = 5.36, *p* = 0.008): at t0, the integrity index was significantly lower in the COVID group than in the control group (t0: m = 0.30 [± 0.15] vs. m = 0.41 [± 0.1]; t(59) = −2.88, *p* = 0.006) and increased significantly in the COVID group from t0 to t1 (m = 0.38 [± 0.17]; t(30) = −3.121, *p* = 0.012) and t2 (m = 0.42 [± 0.22]; t(30) = −5.836, *p* < 0.001).

### Comparison of serological parameters between the two cohorts

Independent of group affiliation, haemoglobin levels changed significantly over time (F [2, 116] = 10.839, *p* < 0.001): there was a significant decrease between t0 and t2 for both groups (control: t0: m = 14.47 [± 1.58] g/dl; t2: m = 14.20 [± 1.59] g/dl, t(29) = 2.711, *p* = 0.033 and COVID: t0: m = 14.32 [± 1.66] g/dl, t2: m = 13.83 [± 1.60] g/dl, t(29) = 3.849, *p* = 0.002). There was a significant interaction for leukocyte count (F [2, 116] = 28.318, *p* < 0.001): at t0, the leukocyte count was significantly lower in the COVID group (t0: m = 5.50 [± 1.75] 10^˄^3/µl) than in the control group (t0: m = 7.90 [± 2.06] 10^˄^3/µl; t(59) = −4.895, *p* < 0.001). Between t0 and t2, there was a highly significant increase in leukocytes in the COVID group (t0: m = 5.50 [± 1.75] 10^˄^3/µl; t2: m = 7.38 [± 2.4] 10^˄^3/µl; t(29) = −7.426, *p* < 0.001), and the increase in leukocytes between t0 and t1 (t1: m = 6.73 [± 2.34] 10^˄^3/µl; t(30) = −4.204, *p* < 0.001) and t1 and t2 was also significant (t(29) = −2.948, *p* = 0.019). In the control group, there was a significant decrease in leukocytes between t0 (m = 7.90 [± 2.06] 10^˄^3/µl) and t2 (m = 7.05 [± 1.87] 10^˄^3/µl; t(29) = 3.071, *p* = 0.014). The changes in leukocytes were within the reference range for leukocytes in both groups. Similar to leukocytes, a significant interaction was observed for neutrophils (F [2, 116] = 17.74, *p* < 0.001): at both t0 (t(59) = −5.141, *p* < 0.001) and t1 (t(59) = −2.245, *p* = 0.029), neutrophils were significantly lower in the COVID group (t0: m = 2.93 ([± 1.21] 10^˄^3/µl; t1: m = 3.69 [± 1.54] 10^˄^3/µl) than in the control group (t0: m = 4.89 [± 1.75] 10^˄^3/µl; t1: m = 4.56 [± 1.77] 10^˄^3/µl). In both groups, there was a significant change in the number of neutrophils between t0 and t2: a significant increase in the COVID group (t2: m = 4.10 [± 1.74] 10^˄^3/µl; t(29) = −4.604, *p* < 0.001) and a significant decrease in the control group (t2: m = 4.01 [± 1.30] 10^˄^3/µl; t(29) = 3.041, *p* = 0.015). A significant interaction was also observed for platelets (F [2, 116] = 18.619, *p* < 0.001): especially at t0 (t(59) = −4.857, *p* < 0.001), but also at t1 (t(59) = −2.071, *p* = 0.042), platelets in the COVID group were significantly lower (t0: m = 222.36 [± 58.64] 10^˄^3/µl; t1: m = 281.97 [± 62.8] 10^˄^3/µl) than in the control group (t0: m = 304.13 [± 71.83] 10^˄^3/µl; t1: m = 322.00 [± 81.06] 10^˄^3/µl). Between t0 and t1 (t(30) = −6.812, *p* < 0.001) and t0 and t2 (t2: m = 294.30 [± 79.19] 10^˄^3/µl; t(29) = −8.293, *p* < 0.001), there was a significant increase in platelets in the COVID group. The mean changes in platelets were also within the reference range in both groups.

**Fig. 2:**
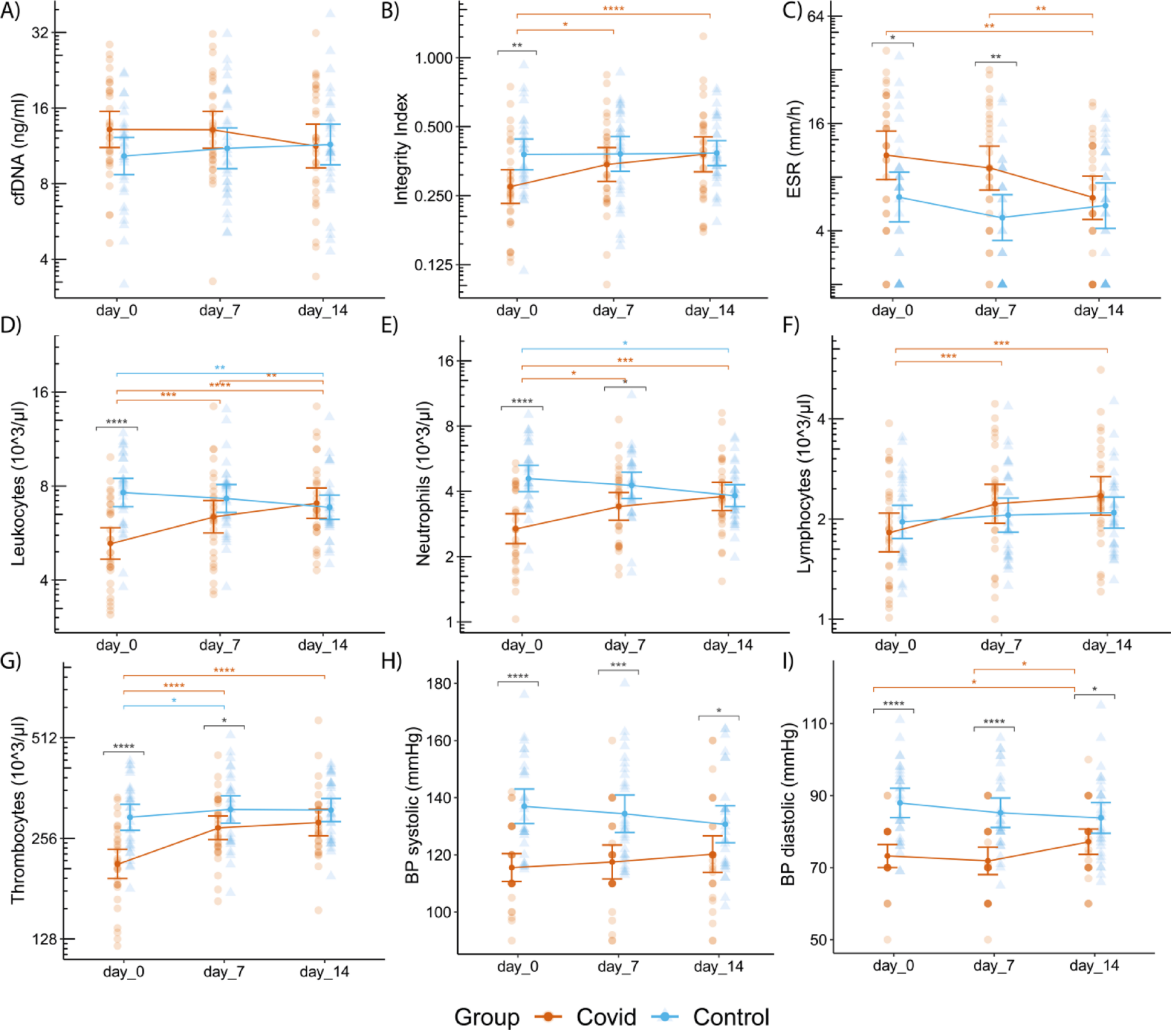
Temporal dynamics of various blood and clinical parameters in COVID patients (brown) and controls (blue) at days 0, 7, and 14. (A) represents cell-free DNA (cfDNA, ng/ml), (B) represents cfDNA integrity index, (C) represents the erythrocyte sedimentation rate (ESR, mm/h), (D) represents Leukocytes (10^3/µl), (E) represents Neutrophils (10^3/µl), (F) represents Lymphocytes (10^3/µl), (G) represents Thrombocytes (10^3/µl), (H) represents the systolic blood pressure (BP systolic; mmHg), (I) represents the diastolic blood pressure (BP diastolic, mmHg). Data are shown as individual values with mean ± 95% confidence interval. Statistical significance between time points and between groups (post-hoc) was indicated only when the preceding ANOVA yielded a significant result. Significance levels: *p < 0.05; **p < 0.01; ***p < 0.001

For full results, see Table 2; Fig. 2.

**Table 2:** Results of the (non-parametric) ANOVAs comparing various parameters between controls and COVID patients for the three time points

Parameter	Group							Results ANOVA/non-parametric ANOVA#					
		Day 0		Day 7		Day 14		Group		Time point		Group*time point	
		mean	sd	mean	sd	mean	sd	F _(1, 59)_	*p*-value	F _(2, 118)_	*p*-value	F _(2, 118)_	*p*-value
cfDNA (90 bp) [ng/ml]	COVID (*n* = 31)	14.45	6.24	14.46	6.57	12.94	6.66	1.538	0.22	0.549	0.579	2.907	0.059
	Control (*n* = 30)	11.32	4.79	12.53	6.67	12.93	7.02						
Integrity (90 bp/222 bp)	COVID (*n* = 31)	0.30	0.15	0.38	0.17	0.42	0.22	2.289	0.136	F _(1.76, 103.93)_ = 6.281	*0.004*	F _(1.76, 103.93)_ = 5.36	*0.008*
	Control (*n* = 30)	0.41	0.17	0.42	0.18	0.41	0.14						
CRP [mg/l]#	COVID (*n* = 31)	11.07	13.92	4.98	6.61	2.35 (*n* = 30)	2.01	F _(1, 58.97)_ = 0.008	0.927	F _(2, 117.2)_ = 12.703	*< 0.001*	F _(2, 117.2)_ = 0.670	0.513
	Control (*n* = 30)	10.45	17.35	4.48	4.57	4.48	6.44						
ESR [mm/hour]#	COVID (*n* = 31)	14.23	9.83	11.68	8.19	7.87 (*n* = 30)	5.36	5.699	0.02	F _(2, 116.19)_ = 11.726	*< 0.001*	F _(2, 116.19)_ = 10.02	*< 0.001*
	Control (*n* = 30)	8.9	8.46	6.47	5.51	7.21 (*n* = 29)	4.97						
Haemoglobin [gdl]	COVID (*n* = 31)	14.32	1.66	14.11	1.62	13.83 (*n* = 30)	1.60	F _(1, 58)_ = 0.439	0.51	F _(2, 116)_ = 10.839	*< 0.001*	F _(2, 116)_ = 0.777	0.462
	Control (*n* = 30)	14.47	1.59	14.38	1.61	14.20	1.59						
Leukocytes [10^˄^3/µl]	COVID (*n* = 31)	5.5	1.75	6.73	2.34	7.38 (*n* = 30)	2.4	F _(1, 58)_ = 5.777	0.019	F _(2, 116)_ = 7.402	*< 0.001*	F _(2, 116)_ = 28.318	*< 0.001*
	Control (*n* = 30)	7.9	2.06	7.58	2.2	7.05	1.87						
Monocytes (absolute) [10^˄^3/µl]	COVID (*n* = 31)	0.50	0.19	0.52	0.19	0.56 (*n* = 30)	0.17	F _(1, 58)_ = 4.729	0.034	F _(1.67, 96.64)_ = 1.83	0.172	F _(1.67, 96.64)_ = 6.139	*0.005*
	Control (*n* = 30)	0.7	0.26	0.57	0.21	0.60	0.25						
Lymphocytes [10^˄^3/µl]	COVID (*n* = 31)	1.95	0.73	2.37	0.85	2.5 (*n* = 30)	0.95	F _(1, 58)_= 0.257	0.614	F _(2, 116)_= 12.815	*< 0.001*	F _(2, 116)_= 4.728	*0.011*
	Control (*n* = 30)	2.05	0.65	2.16	0.74	2.18	0.7						
Thrombocytes [10^˄^3/µl]	COVID (*n* = 31)	222.36	58.64	281.97	62.8	294.3 (*n* = 30)	79.19	F _(1, 58)_ = 9.455	0.003	F _(2, 116)_= 39.557	*< 0.001*	F _(2, 116)_= 18.619	*< 0.001*
	Control (*n* = 30)	304.13	71.83	322	81.06	317.83	66.54						
Neutrophils (absolute) [10^˄^3/µl]	COVID (*n* = 31)	2.93	1.21	3.69	1.54	4.1 (*n* = 30)	1.74	F _(1, 58)_ = 9.583	0.003	F _(2, 116)_= 2.553	0.082	F _(2, 116)_= 17.74	*< 0.001*
	Control (*n* = 30)	4.89	1.75	4.56	1.77	4.01	1.3						
Eosinophils (absolute) [10^˄^3/µ]#	COVID (*n* = 31)	0.1	0.08	0.15	0.08	0.17 (*n* = 30)	0.1	5.493	0.022	F _(2, 117.1)_ = 5.317	*0.006*	F _(2, 117.1)_ = 11.112	*< 0.001*
	Control (*n* = 30)	0.22	0.13	0.2	0.12	0.2	0.14						
Basophils (absolute) [10^˄^3/µl)#	COVID (*n* = 31)	0.02	0.02	0.03	0.02	0.04 (*n* = 30)	0.02	35.694	< 0.001	F _(2, 117.2)_= 11.218	*< 0.001*	F _(2, 117.2)_= 8.933	*< 0.001*
	Control (*n* = 30)	0.05	0.02	0.06	0.03	0.05	0.03						
Heart rate [beats per minute]	COVID (*n* = 31)	78	12.16	76.26	12.03	78.43 (*n* = 30)	11.46	F _(1, 57)_ = 0.037	0.848	F _(2, 114)_= 1.322	0.271	F _(2, 114)_= 0.385	0.681
	Control (*n* = 30)	78.97	13.66	75.87	10.75	76.76 (*n* = 29)	11.58						
BP systolic [mmHg]	COVID (*n* = 31)	115.55	13.3	117.52	16.16	120.26	17.45	19.566	< 0.001	0.121	0.886	5.71	*0.004*
	Control (*n* = 30)	137	16.16	134.4	17.57	130.7	17.36						
BP diastolic [mmHg]#	COVID (*n* = 31)	73.23	8.71	71.87	10.4	77.19	9.6	23.555	< 0.001	3.826	*0.024*	9.245	*< 0.001*
	Control (*n* = 30)	87.97	10.93	85.23	10.95	83.8	11.39						
Oxygen saturation [percent]#	COVID (*n* = 31)	97.55	1.15	97.97	1.8	97.97 (*n* = 30)	1.27	F _(1, 58)_ = 12.017	< 0.001	F _(2, 116.9)_= 7.190	*0.001*	F _(2, 116.8)_= 1.358	0.261
	Control (*n* = 30)	98.37	0.85	98.33	1.35	98.76 (*n* = 29)	0.64						

### Comparison of clinical parameters between the two cohorts

A significant interaction was observed for both systolic (F [2, 118] = 5.71, *p* = 0.004) and diastolic blood pressure (BP) (F [2, 118] = 9.245; *p* < 0.001). Systolic and diastolic BP were significantly lower in the COVID group than in the control group at all three time points: systolic BP: t0: m = 115.55 (± 13.30) mmHg vs. 137.0 (± 16.16) mmHg (t(59) = −5.767, *p* < 0.001); t1: m = 117.52 (± 16.16) mmHg vs. 134.4 (± 17.57) mmHg (t(59) = −4.012, *p* < 0.001); t2: 120.26 (± 17.45) mmHg vs. 130.7 (± 17.36) mmHg (t(59) = −2.421, *p* = 0.019); diastolic BP: t0: m = 73.23 (± 8.71) mmHg vs. 87.97 (± 10.93) mmHg (W = 147, *p* < 0.001); t1: m = 71.87 (± 10.4) mmHg vs. 85.23 (± 10.95) mmHg (W = 166, *p* < 0.001); t2: m = 77.19 (± 9.6) mmHg vs. 83.80 (± 11.39) mmHg (W = 321, *p* = 0.037). In the COVID group there was a significant increase in diastolic blood pressure between t1 and t2 (W = 51.5, *p* = 0.012), whereas in the control group there was a significant decrease in systolic blood pressure between t0 and t2 (t(29) = 2.795, *p* = 0.02). Further details can be found in Table 2; Fig. 2.

### Comparison of symptoms between the two cohorts

**Fig. 3:**
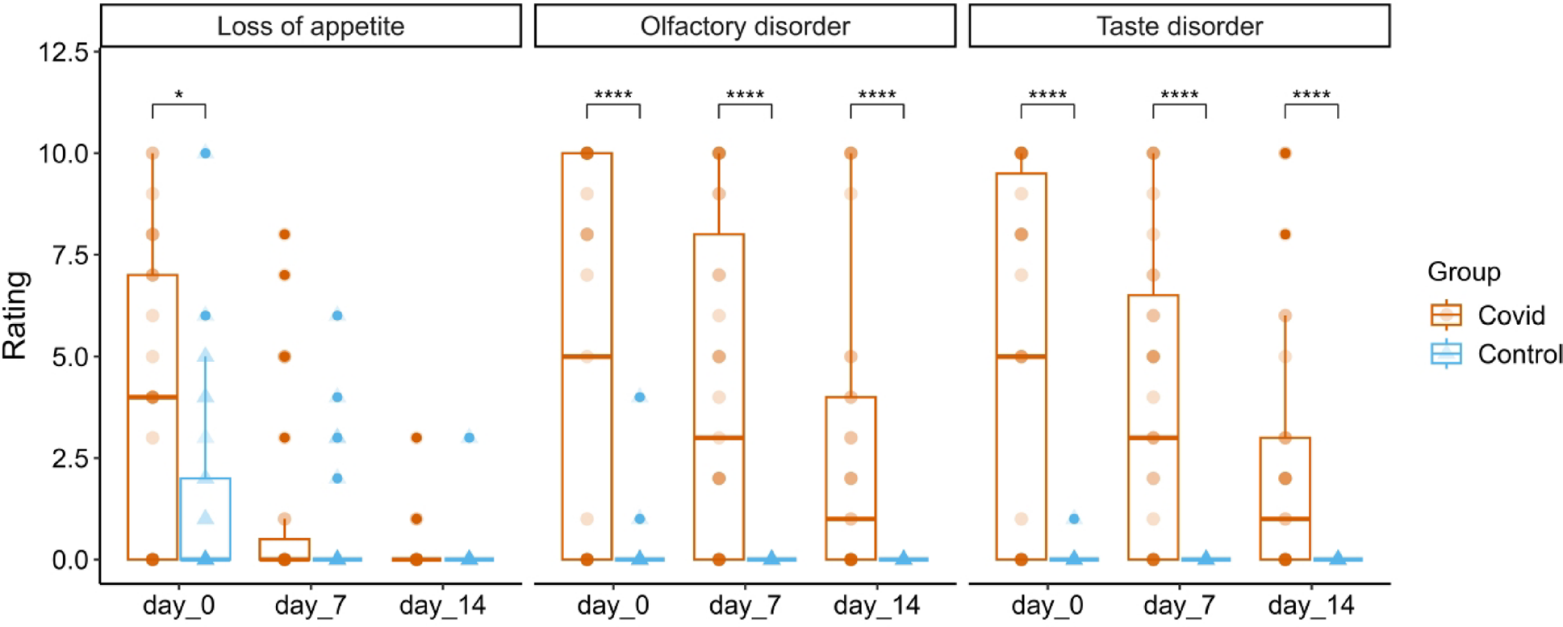
Symptom severity ratings of the symptoms loss of appetite, olfactory disorder and taste disorder over time in COVID patients (brown) and controls (blue) at days 0, 7, and 14. Box plots represent the median, interquartile range and individual values. Loss of appetite, olfactory disorder and taste disorder yielded statistically significant results in post-hoc testing with higher symptom scores in COVID-19 patients compared to controls. Significance levels: *p < 0.05; **p < 0.01; ***p < 0.001

A number of symptoms showed significant interactions. However, post-hoc Wilcoxon tests indicated group differences for loss of appetite, olfactory disorder and taste disorder (see Fig. 3 and the table in the Supplement).

### Correlation of CfDNA levels with measured laboratory parameters, clinical parameters and symptom severity

There was a weak correlation between cfDNA and haematological parameters in the control group including leukocyte count, neutrophil count, basophil count, monocyte count, as well as systolic and diastolic blood pressure, which were not detectable in the COVID group (see Table 3).

**Table 3:** Spearman correlation between CfDNA 90 and laboratory and clinical parameters in the COVID and control group

Parameters	Control		COVID	
	Correlation	*p*-value	Correlation	*p*-value
Haemoglobin [g/dl]	0.073	0.488	0.127	0.218
Leukocytes [10^˄^3/µl**]**	0.257	0.002**	−0.068	0.511
Basophils (absolute) [10^˄^3/µl]	0.284	0.001***	−0.248	0.056
Lymphocytes [10^˄^3/µl]	0.040	0.100	−0.017	0.826
Neutrophils (absolute) [10^˄^3/µl]	0.235	0.017*	−0.089	0.379
Monocytes (absolute) [10^˄^3/µl]	0.219	0.010**	0.163	0.177
Eosinophils (absolute) [10^˄^3/µl]	0.130	0.019*	−0.144	0.086
CRP [mg/l]	0.130	0.121	0.072	0.824
ESR [mm/hour]	0.226	0.017*	−0.128	0.820
Diastolic blood pressure [mmHg]	0.216	0.203	0.084	0.555
[mmHg]	0.330	0.006**	0.238	0.007**

## Discussion

To the best of our knowledge, this prospective cohort study is the first in Germany —and one of the first internationally— to investigate cfDNA levels and fragmentation patterns (integrity index) in outpatients with influenza-like symptoms with and without SARS-CoV-2 detection directly recruited and monitored in GP practices under primary care conditions. Unlike most previous studies that focused on hospitalised or severely ill patients [26, 27, 29], our study captures the biological response to SARS-CoV-2 in a mild, ambulatory context.

We showed that cfDNA levels from outpatients with flu-like symptoms and detection of SARS-CoV-2 do not significantly differ from those in patients with flu-like symptoms who test negative for SARS-CoV-2. However, the cfDNA integrity index was significantly lower at baseline in the COVID group, indicating a higher degree of fragmentation. Despite the partly significant differences in the laboratory parameters (e.g. leukocytes, neutrophils, platelets), the values remained within the reference range of the individual laboratory values. Furthermore, COVID patients had significantly lower systolic and diastolic blood pressure levels compared to the control group across all three time points. In terms of symptoms, COVID patients reported significantly more olfactory and gustatory dysfunction at all three time points, and a significantly greater loss of appetite at baseline.

While most studies on cfDNA have focused on hospitalised and often critically ill COVID-19 patients, only a few have examined cfDNA levels in outpatients with mild disease. These studies showed that cfDNA levels correlate with disease severity, as defined by the WHO progression scale: patients with severe COVID-19 progression, those admitted to intensive care and those who die during the course of the disease exhibit significantly higher cfDNA levels than those with mild to moderate illness [26, 27, 29, 37, 38]. Our results in a primary care setting support and extend these observations. In our cohort of outpatients with mild COVID-19, cfDNA levels were low and did not differ significantly from those observed in patients with flu-like symptoms caused by other respiratory pathogens. However, the significantly lower cfDNA integrity index observed in the COVID-19 group at baseline suggests a distinct fragmentation pattern of cfDNA release. The cfDNA integrity index is calculated as the ratio of long to short DNA fragments—in this study, 222 bp to 90 bp amplicons. A lower integrity index is typically associated with increased apoptosis, where DNA is cleaved into uniformly short fragments. In contrast, a higher integrity index may indicate necrosis or neutrophil extracellular trap formation (NETosis), both of which release longer and more variable cfDNA fragments into circulation [28]. Thus, the lower integrity index observed in the COVID-19 group may reflect apoptosis-driven immune or tissue responses that are already detectable in the early, mild phase of infection—even in the absence of markedly elevated total cfDNA levels.

This interpretation is supported by the haematological findings. At baseline, patients in the COVID-19 group had significantly lower leukocyte, neutrophil and platelet counts compared with controls. These reductions may reflect peripheral consumption or sequestration of immune cells, or early apoptosis of circulating immune cells—both of which are consistent with cfDNA release through apoptotic pathways [39]. The combination of a lower cfDNA integrity index and cytopenia across multiple cell lines suggests that even mild SARS-CoV-2 infections can induce distinct and measurable immune activation and cell turnover, despite the absence of severe clinical symptoms.

These patterns are also known from other viral infections such as influenza and infectious mononucleosis, where similar blood count changes have been observed [40–42]. In contrast to severe COVID-19, where lymphopenia is a well-established marker of poor prognosis [40, 43–45], lymphocyte counts did not differ significantly between the groups in our study, reflecting the mild clinical presentation in all participants. Although C-reactive protein levels were not significantly different between the two groups, the erythrocyte sedimentation rate (ESR) was significantly higher in the COVID group than in the control group at t0 and t1, indicating a low-grade inflammatory response. Despite these partly significant differences in the laboratory parameters, the differences were within the reference range of the individual laboratory values, underlining the subclinical nature of these alterations in COVID-19. These subtle yet measurable molecular and haematological changes in mild COVID-19 cases highlight the potential of cfDNA—particularly the integrity index—as an early biomarker for identifying patients who may require closer monitoring. In situations where hospital bed capacity is limited due to a high number of infections, especially in winter, early risk stratification becomes critical. It would therefore be important for GPs to be able to assess early which COVID outpatients are at higher risk. cfDNA could be a suitable prognostic marker: outpatients with COVID-19 who have high cfDNA levels and low integrity indices might benefit from closer monitoring, earlier hospital admission and earlier antiviral intervention (e.g. Paxlovid^®^). So far, however, these considerations have remained theoretical, as there is currently no way of measuring the cfDNA level in a rapid, simple and cost-effective way.

In terms of clinical characteristics, blood pressure differed significantly between the two groups in our study: at t0, mean systolic blood pressure in the COVID group was almost 22 mmHg lower than in the control group. Although the mean systolic blood pressure in the COVID-19 patients increased during the observation period, it remained almost 10 mmHg lower than in the control group at t2. However, systolic blood pressure in the COVID group did not fall below 100 mmHg, a threshold associated with severe outcomes in previous studies [46, 47]. Participants did not spontaneously report symptoms of orthostatic hypotension (OH). However, an observational cross-sectional study of non-critically ill COVID-19 patients reported that even mildly ill COVID patients experienced significantly more episodes of OH than controls [48]. This suggests that GPs should proactively ask about symptoms of OH and recommend preventive measures (slow changes of position, adequate fluid intake, etc.).

Overall, symptom severity was low in both groups. Only the non-specific symptom general weakness reached a notable mean score of all symptoms at t0 in the COVID group at 5.03. In addition to the well-known more pronounced olfactory and gustatory dysfunction in COVID-19 patients [49, 50], which was also seen in our study at all three time points, the COVID-19 patients reported a significantly greater loss of appetite at t0 than the control group. In contrast to the findings of previous studies [51, 52], our research did not identify a statistically significant difference in the severity of sore throat symptoms between the two groups, indicating the dynamic nature of clinical presentation in COVID-19 depending on the circulating variants.

In line with previous studies [53], our results suggest a weak correlation between cfDNA and haematological parameters in the control group. This correlation was not found in the COVID group, but the reasons for this require further investigation.

### Strength and limitations

A key strength of the study is that only patients with mild symptoms of a flu-like infection (with and without the presence of SARS-CoV-2) from general practices were included. The two groups were comparable in terms of gender, age and pre-existing conditions. There were no known serious pre-existing medical conditions that could have affected cfDNA levels in the study participants. Data could be collected from all study participants over the two-week observation period, which in the case of the COVID patients included home visits due to the isolation requirements in place at the time.

The vaccination status of the patients could not be reliably documented and was therefore not included in the data analysis, as vaccination records were often unavailable at the time of enrolment and visits. In the control group, only the presence of SARS-CoV-2 infection was tested; other respiratory pathogens such as influenza were not tested for cost reasons. Based on the weekly data published by the Robert Koch Institute on circulating respiratory pathogens and the very low prevalence of influenza during the study period [3], pathogens such as influenza that could have had an influence on cfDNA levels comparable to SARS-CoV-2 can be excluded.

## Conclusions

In contrast to patients with severe COVID-19, we were able to show that there was no significant difference in cfDNA levels between patients with a mild flu-like infection and SARS-CoV-2 detection and those without a SARS-CoV-2 infection. However, the significantly lower cfDNA integrity index in the COVID group indicates a distinct fragmentation pattern, likely reflecting early apoptotic processes. These findings support the potential of cfDNA, particularly the integrity index, as a prognostic biomarker for the early identification of patients at risk for a severe course in the general practitioner’s office. For this to be possible, however, a low-threshold and cost-effective method of determining cfDNA levels must be developed. At present, the only option for GPs is to perform a differential blood count to detect early lymphopenia, which appears to correlate with severe disease in other studies and did not occur in our population of mildly ill COVID outpatients. In addition, routine monitoring of blood pressure may help detect early signs of clinical instability, as a systolic blood pressure below 100 mmHg has been linked to adverse outcomes in COVID-19.

## Supplementary Information

Supplementary Material 1.

## Data Availability

The study principal investigator and the co-investigators have access to the full study data and materials. The authors are willing to share the individual-level study data after completion and publication of primary and secondary analyses.
