# Supplementary material for: CfDNA as a surrogate marker for COVID-19 severity in patients with influenza-like symptoms with and without SARS-CoV-2 infection in general practice: a prospective cohort study
Source: Virol J. 2025 Dec 1;22:390. doi: 10.1186/s12985-025-03016-x (PMC12667079; doi:10.1186/s12985-025-03016-x)
Supplement: Supplementary file 1 — Supplementary Material 1. [file 12985_2025_3016_MOESM1_ESM.docx]

| **Table 4:** Results of the non-parametric ANOVAs comparing various symptoms between controls and COVID patients for the three time points | | | | | | | | | | | | | | | |
| --- | --- | --- | --- | --- | --- | --- | --- | --- | --- | --- | --- | --- | --- | --- | --- |
|  | | | | | | | | **Results non-parametric ANOVA** | | | | | | | |
| **Parameter** | **Group** | **Day 0** | | **Day 7** | | **Day 14** | | **Group** | | | **Timepoint** | | | **Group*Timepoint** | |
|  |  | **mean** | **sd** | **mean** | **sd** | **mean** | **sd** | **F _(1, 59)_** | **p-value** | **F _(2, 118)_** | | **p-value** | **F _(2, 118)_** | | **p-value** |
| General weakness | Covid (n=31) | 5.03 | 3.50 | 2.71 | 3.13 | 1.29 | 2.07 | 0.475 | 0.493 | 29.398 | | *<0.001* | 2.214 | | 0.114 |
|  | Control (n=30) | 4.13 | 2.65 | 2.17 | 2.57 | 1.47 | 2.22 |  |  |  |  |  |  |  |  |
| Loss of appetite | Covid (n=31) | 3.61 | 3.57 | 1.13 | 2.31 | 0.16 | 0.58 | 13.910 | *<0.001* | 28.785 | | *<0.001* | 17.688 | | *<0.001* |
|  | Control (n=30) | 1.43 | 2.46 | 0.60 | 1.48 | 0.10 | 0.55 |  |  |  |  |  |  |  |  |
| Nausea | Covid (n=31) | 1.00 | 2.48 | 1.00 | 2.63 | 0.42 | 1.54 | 3.888 | 0.053 | 1.962 | | 0.145 | 1.342 | | 0.265 |
|  | Control (n=30) | 0.20 | 0.93 | 0.17 | 0.91 | 0.13 | 0.73 |  |  |  |  |  |  |  |  |
| Chest pain | Covid (n=31) | 1.45 | 2.67 | 0.32 | 1.01 | 0.10 | 0.54 | 28.114 | *<0.001* | 6.856 | | *0.002* | 6.420 | | *0.002* |
|  | Control (n=30) | 0.70 | 1.47 | 0.13 | 0.73 | 0.20 | 0.76 |  |  |  |  |  |  |  |  |
| Diarrhea | Covid (n=31) | 0.81 | 1.72 | 0.48 | 1.26 | 0.10 | 0.40 | 0.033 | 0.855 | 4.140 | | *0.018* | 1.205 | | 0.303 |
|  | Control (n=30) | 0.60 | 1.99 | 0.33 | 1.06 | 0.13 | 0.73 |  |  |  |  |  |  |  |  |
| Fever | Covid (n=31) | 1.00 | 2.18 | 0.26 | 1.26 | 0.00 | 0.00 | 7.057 | *0.010* | 11.237 | | *<0.001* | 2.099 | | 0.127 |
|  | Control (n=30) | 0.70 | 1.71 | 0.20 | 1.10 | 0.13 | 0.73 |  |  |  |  |  |  |  |  |
| Odor disorder | Covid (n=31) | 4.71 | 4.69 | 4.13 | 4.01 | 2.61 | 3.58 | **F _(1, 58.97_**_)_= 27.913 | *<0.001* | **F _(2, 117.36_**_)_= 12.314 | | *<0.001* | **F _(2, 117.35_**_)_= 9.756 | | *<0.001* |
|  | Control (n=30) | 0.17 (n=29) | 0.76 | 0.00 | 0.00 | 0.00 | 0.00 |  |  |  |  |  |  |  |  |
| Taste disorder | Covid (n=31) | 4.81 | 4.36 | 3.90 | 3.63 | 2.13 | 3.00 | **F _(1, 58.98_**_)_= 31.875 | *<0.001* | **F _(2, 117.30_**_)_= 23.341 | | *<0.001* | **F _(2, 117.31_**_)_= 21.626 | | *<0.001* |
|  | Control (n=30) | 0.03 (n=29) | 0.19 | 0.00 | 0.00 | 0.00 | 0.00 |  |  |  |  |  |  |  |  |
| Sore throat | Covid (n=31) | 2.03 | 2.74 | 0.58 | 1.41 | 0.26 | 1.00 | **F _(1, 58.97_**_)_= 4.045 | *0.049* | **F _(2, 117.31_**_)_= 27.212 | | *<0.001* | **F _(2, 117.35_**_)_= 6.694 | | *0.002* |
|  | Control (n=30) | 3.03 (n=29) | 2.71 | 1.43 | 2.57 | 0.83 | 1.80 |  |  |  |  |  |  |  |  |
| Cough with sputum | Covid (n=31) | 2.26 | 3.10 | 1.68 | 2.61 | 0.45 | 1.15 | **F _(1, 58.99_**_)_= 0.004 | 0.953 | **F _(2, 117.21_**_)_= 3.058 | | 0.051 | **F _(2, 117.23_**_)_= 2.024 | | 0.137 |
|  | Control (n=30) | 1.66 (n=29) | 2.72 | 1.53 | 2.42 | 1.13 | 1.98 |  |  |  |  |  |  |  |  |
| Dry cough | Covid (n=31) | 2.52 | 3.10 | 1.90 | 2.95 | 1.00 | 1.65 | **F _(1, 58.95_**_)_= 0.941 | 0.336 | **F _(2, 116.66_**_)_= 7.589 | | *0.001* | **F _(2, 116.69_**_)_= 1.389 | | *0.253* |
|  | Control (n=30) | 2.59 | 2.64 | 2.31 (n=29) | 2.90 | 0.87 | 1.87 |  |  |  |  |  |  |  |  |
| Headache | Covid (n=31) | 4.58 | 3.69 | 1.87 | 2.66 | 1.10 | 2.20 | **F _(1, 58.98_**_)_= 0.013 | 0.910 | **F _(2, 117.26_**_)_= 28.256 | | *<0.001* | **F _(2, 117.28_**_)_= 0.172 | | *0.842* |
|  | Control (n=30) | 4.07 (n=29) | 2.28 | 2.43 | 3.03 | 1.93 | 2.86 |  |  |  |  |  |  |  |  |
| Dyspnea | Covid (n=31) | 1.10 | 2.33 | 0.36 | 1.25 | 0.42 | 1.23 | **F _(1, 58.96_**_)_= 13.889 | *<0.001* | **F _(2, 117.34_**_)_= 0.835 | | 0.437 | **F _(2, 117.38_**_)_= 4.608 | | *0.012* |
|  | Control (n=30) | 0.48 (n=29) | 1.15 | 0.30 | 0.79 | 0.33 | 1.03 |  |  |  |  |  |  |  |  |
| Fatigue | Covid (n=31) | 4.45 | 3.63 | 2.61 | 3.13 | 1.26 | 2.59 | **F _(1, 58.98_**_)_= 0.638 | 0.428 | **F _(2, 116.57_**_)_= 23.239 | | *<0.001* | **F _(2, 116.60_**_)_= 0.441 | | *0.644* |
|  | Control (n=30) | 4.39 (n=29) | 2.74 | 3.20 | 2.64 | 1.73 | 1.98 |  |  |  |  |  |  |  |  |
| Muscle and limb pain | Covid (n=31) | 3.77 | 4.00 | 0.58 | 1.41 | 0.55 | 1.69 | **F _(1, 58.96_**_)_ =14.862 | *<0.001* | **F _(2, 117.43_**_)_= 20.241 | | *<0.001* | **F _(2, 117.39_**_)_= 6.149 | | *0.003* |
|  | Control (n=30) | 2.45 (n=29) | 2.68 | 0.47 | 1.63 | 0.67 | 1.61 |  |  |  |  |  |  |  |  |
| Chills | Covid (n=31) | 2.19 | 3.48 | 0.39 | 1.82 | 0.16 | 0.90 | **F _(1, 58.96_**_)_= 2.377 | 0.129 | **F _(2, 117.41_**_)_= 12.377 | | *<0.001* | **F _(2, 117.41_**_)_= 5.225 | | *0.007* |
|  | Control (n=30) | 1.83 (n=29) | 2.52 | 0.27 | 0.91 | 0.33 | 1.30 |  |  |  |  |  |  |  |  |
| Dyspnea on exertion | Covid (n=31) | 0.19 | 0.48 | 0.13 | 0.34 | 0.13 | 0.34 | 1.109 | 0.297 | 0.363 | | 0.697 | 3.304 | | *0.040* |
|  | Control (n=30) | 0.17 | 0.38 | 0.10 | 0.31 | 0.13 | 0.35 |  |  |  |  |  |  |  |  |
